# TNFR2 as a Potential Biomarker for Early Detection and Progression of CKD

**DOI:** 10.3390/biom13030534

**Published:** 2023-03-15

**Authors:** Irina Lousa, Flávio Reis, Sofia Viana, Pedro Vieira, Helena Vala, Luís Belo, Alice Santos-Silva

**Affiliations:** 1 Associate Laboratory i4HB—Institute for Health and Bioeconomy, Faculty of Pharmacy, University of Porto, 4050-313 Porto, Portugal; 2 UCIBIO—Applied Molecular Biosciences Unit, Laboratory of Biochemistry, Department of Biological Sciences, Faculty of Pharmacy, University of Porto, 4050-313 Porto, Portugal; 3 Institute of Pharmacology & Experimental Therapeutics & Coimbra Institute for Clinical and Biomedical Research (iCBR), Faculty of Medicine, University of Coimbra, 3000-548 Coimbra, Portugal; 4 Center for Innovative Biomedicine and Biotechnology (CIBB), University of Coimbra, 3004-504 Coimbra, Portugal; 5 Clinical Academic Center of Coimbra (CACC), 3000-075 Coimbra, Portugal; 6 Polytechnic Institute of Coimbra, ESTESC-Coimbra Health School, Pharmacy, 3046-854 Coimbra, Portugal; 7 CERNAS, Escola Superior Agrária de Viseu, Instituto Politécnico de Viseu, Campus Politécnico, 3504-510 Viseu, Portugal; 8 Centre for the Research and Technology of Agro-Environmental and Biological Sciences (CITAB), University of Trás-os-Montes e Alto Douro, 5001-801 Vila Real, Portugal

**Keywords:** CKD, inflammation, TNFR1, TNFR2, biomarkers

## Abstract

The inflammatory pathway driven by TNF-α, through its receptors TNFR1 and TNFR2, is a common feature in the pathogenesis of chronic kidney disease (CKD), regardless of the initial disease cause. Evidence correlates the chronic inflammatory status with decreased renal function. Our aim was to evaluate the potential of TNF receptors as biomarkers for CKD diagnosis and staging, as well as their association with the progression of renal lesions, in rat models of early and moderate CKD. We analyzed the circulating levels of inflammatory molecules—tumor necrosis factor-alpha (TNF-α), tumor necrosis factor receptor 1 (TNFR1) and 2 (TNFR2) and tissue inhibitor of metalloproteinase-1 (TIMP-1)—and studied their associations with TNFR1 and TNFR2 renal expression, glomerular and tubulointerstitial lesions, and with biomarkers of renal (dys)function. An increase in all inflammatory markers was observed in moderate CKD, as compared to controls, but only circulating levels of both TNFR1 and TNFR2 were significantly increased in the early disease; TNFR2 serum levels were negatively correlated with eGFR. However, only TNFR2 renal expression increased with CKD severity and showed correlations with the score of mild and advanced tubular lesions. Our findings suggest that renal TNFR2 plays a role in CKD development, and has potential to be used as a biomarker for the early detection and progression of the disease. Still, the potential value of this biomarker in disease progression warrants further investigation.

## 1. Introduction

According to the Global Burden of Disease (GBD) studies, chronic kidney disease (CKD) has emerged as a major cause of global morbidity and mortality, affecting more than 800 million individuals worldwide and imposing major socio-economic costs [1,2]. CKD is an heterogenous condition, with a broad range of underlying etiologies, clinical manifestations and variable progression rates [3].

Inflammation has been implicated in the progression and outcome of CKD, regardless of the initial disease cause. Emerging data suggest an association between biomarkers of inflammation and decreased renal function, as a result of the underlying kidney injury mechanisms [4,5,6]. Therefore, it has been suggested that more sensitive measurements of inflammation could outperform the classical kidney function estimation tools currently used for CKD diagnosis and prognosis [7,8], namely glomerular filtration rate (GFR) and albuminuria assessment. Moreover, the identification of early biomarkers of the inflammatory milieu underlying CKD would allow an early detection and intervention to prevent disease worsening, and reduce the associated socioeconomic costs.

Circulating levels of molecules involved in the tumor necrosis factor alpha (TNF-α) pathway, such as TNF receptors 1 and 2 (TNFR1 and TNFR2), were shown to be increased in CKD, in several cohorts of patients, age-groups and races, and with different disease etiologies [9,10,11,12,13]. Recently, our group reported that the increase in the circulating levels of TNFR2 were associated with renal function decline, suggesting that the TNF-α inflammation pathway reflects disease progression [14]; moreover, TNFR2 appeared to be useful for an early detection of CKD, presenting a significantly higher value, as compared to the control group. The tissue inhibitor of metalloproteinase-1 (TIMP-1), an extracellular matrix remodeling regulator, is upregulated in renal interstitial fibrosis [15] and has been linked to the occurrence of renal inflammation in CKD [16]. However, in a mice model with overload proteinuria, the severity of interstitial fibrosis was not changed when the TIMP-1 gene was knocked out [17].

The multiple drug therapies (such as statins, antihypertensive, diuretics, etc.), as well as the prevalence and complexity of comorbidities in CKD patients, represent a constraint when analyzing inflammatory parameters in these populations. Moreover, most of the studies on inflammation biomarkers are limited to individuals with CKD without a biopsy confirmation of cause [9,18,19,20], and therefore, we cannot infer whether those biomarkers provide specificity for kidney histopathologic lesions. Despite being invasive procedures with associated risks, kidney biopsies provide insights on glomerular and tubulointerstitial histology, which are associated with the risk of CKD progression and death [21]. Due to the limitations and ethical issues for these procedures, cellular and tissue renal studies, using appropriate animal models, would be useful to evaluate the sensitivity and specificity of new, potential, earlier and more sensitive CKD biomarkers, than the traditional ones, by searching for correlations between the circulating levels of a biomarker, its renal cell expression and associated kidney lesions. The characterization of novel CKD biomarkers and its association with underlying histopathologic lesions might allow the identification of non-invasive early disease and/or prognostic biomarkers, and also provide information on the clinical phenotyping of kidney diseases.

Most published animal studies highlight the importance of TNF-α and its receptors in the development of kidney diseases by demonstrating reduced disease activity with a TNF-α blockade or using knockout models [22,23,24,25]; however, their association with clinical and histopathological findings has not been subject of investigation.

In this study, we used rat models of early and moderate CKD, induced by nephrectomy, to characterize inflammatory biomarkers in CKD staging. We measured the serum levels of TNF-α, TNFR1 and TNFR2, and their renal cell expression, and performed kidney histological studies, in rats with different disease staging. Since chronic inflammation has been implicated in the progression and outcome of CKD, we hypothesized that these inflammatory biomarkers might reflect early renal cell and histological changes and, therefore, might be useful for early CKD detection and/or for staging.

## 2. Materials and Methods

### 2.1. Animal Welfare and Experimental Design

All rodent experiments were conducted according to the National and European Community Council directives on animal care. The project received approval (7/2020) from the local Organization Responsible for Animal Welfare (ORBEA, from Coimbra Institute for Clinical and Biomedical Research, Faculty of Medicine, University of Coimbra) and complied with the Animal Care National and European Directives and with ARRIVE guidelines [26].

Male Wistar rats (Charles River Laboratories, Barcelona, Spain) were housed two per cage and maintained in rooms with a 12 h light/dark cycle, under a controlled temperature (22 °C) and humidity (50–60%). Animals received free access to standard rat laboratory chow (4RF21 Mucedola, Milan, Italy) and water. Body weight was monitored every week. After an adaptation period, rats at 12 weeks of age were randomly divided in three groups (sham, mild CRF and moderate CRF) and submitted to a 5-week protocol.

Mild chronic renal failure (Mild CRF, n = 8) was induced by the complete removal of the left kidney (day 0), and one week later (day 7), a right flank incision was performed without renal mass reduction. Moderate chronic renal failure (moderate CRF, n = 7) was induced by a two-step 5/6 nephrectomy, with surgical incision of both poles of the left kidney (2/3 nephrectomy) at day 0, and, one week later (day 7), the complete removal of the right kidney. The sham operated group (n = 8) was subjected to surgical process without kidney mass reduction (days 0 and 7) and used as control.

To perform the surgical procedures, rats were subjected to intraperitoneal anesthesia with 75 mg/Kg ketamine (Nimatek, Eurovet Animal Health BV, Bladel, The Netherlands) and 1 mg/Kg medetomidine (Sedator, Dechra, Barcelona, Spain). In the post-surgical period, the animals were kept on recovery blankets with a controlled temperature, and analgesia (0.05 mg/Kg of buprenorphine, Bupaq^®^, Richter Pharma AG, Austria). Anesthesia was reversed with 2.5 mg/Kg atipamezole (Revazol, Dechra, Barcelona, Spain).

### 2.2. Samples Collection

Rats were enclosed in metabolic cages for 24 h before the sacrifice (day 35), with free access to laboratory chow and water, for the collection of 24 h urine. Urine volume and water consumption were recorded, and urine aliquots were stored at −80 °C.

At the end of the protocol (day 36), rats were sacrificed using an overdose of intraperitoneal 80 mg/Kg pentobarbital (Sigma-Adrich, Saint-Louis, MO, USA), and blood was collected through cardiac puncture into tubes with K_3_EDTA or without anticoagulant for hematological and biochemical studies. Serum and plasma aliquots were immediately stored at −80 °C until they were assayed. Kidneys were immediately removed, cleaned and weighed, and stored for further analysis.

### 2.3. Hematological and Biochemical Analysis

Red blood cells (RBC), leukocyte, platelet and reticulocytes counts, hemoglobin concentration and hematocrit values were evaluated using an automated cell counter (HORIBA ABX, Amadora, Portugal). The reticulocyte production index (RPI) was calculated as previously described [27].

Blood urea nitrogen (BUN) and serum creatinine were evaluated using validated automated methods and equipment (Hitachi 717 Chemical analyser, Roche Diagnostics, Basel, Switzerland). The urinary levels of creatinine and urea were analyzed in the 24 h urine using automatic methods (Cobas Integra 400 Plus, Roche Diagnostics, Basel, Switzerland). GFR, urea and creatinine clearances were calculated as previously described by Pestel et al. [28].

Serum or plasma biomarkers levels were measured with rat-specific ELISA kits, in accordance with the manufacturer’s instructions (TIMP-1 Rat ELISA Kit and Rat TNF-alpha ELISA Kit, Life Technologies, Carlsband, CA, USA; Rat TNFRSF1A ELISA Kit and Rat TNFRSF1B ELISA kit, MyBiosource, San Diego, CA, USA).

### 2.4. Histopathological Analysis

Renal tissue samples were formalin-fixed, embedded in paraffin wax, and 4 µm thick sections were stained with Periodic acid–Shiff (PAS) (Sigma Aldrich, Saint Louis, MO, USA). All samples were examined under light microscopy, using a Zeiss microscope Axioplan 2 (Carl Zeiss Microscopy, LLC, NY, USA) and images were captured using a digital microscope camera (Leica DFC450, Leica Microsystems, Wetzlar, Germany) at 400× magnification. Mild and advanced glomerular and tubulointerstitial lesions were identified and evaluated in the total tissue of the slide. The severity of lesions was semi-quantitatively rated according to the extension occupied by the lesion (assessed % over total cortical area or glomeruli affected): 0—absent/normal (<5%); 1—mild (5–25%); 2—moderate (25–50%); 3—severe (>50%). Glomerular hypertrophy was analyzed by measuring the area of ten cortical glomeruli and ten juxtamedullary glomeruli, randomly selected, in each rat (ImageJ processing software, U.S. National Institute of Health, Bethesda, MD, USA). The final score of each lesion was obtained after averaging the individual scores of the animals [29]. Renal pathology evaluation was confirmed by a senior pathologist in a blinded fashion.

### 2.5. Immunohistochemistry Analysis

Renal paraffin sections were used for immunohistochemical staining, after incubation with xylene and rehydration with graded ethanol series to water. A mouse- and rabbit-specific horseradish peroxidase (HRP)/diaminobenzidine (DAB) detection kit (ab80436, Abcam Inc, Cambridge, UK) was used according to the manufacturer’s protocol. To retrieve antigen exposure, samples were treated with 10 mM citrate buffer solution at 95 °C, for 30 min. Sections were incubated with primary antibodies at 4 °C overnight in a humidified chamber. Primary antibodies were used for detection of TNF-α (dilution 1:100, PA1-40281), TNFR1 (dilution 1:1000, PA5-95585) and TNFR2 (dilution 1:800, MA5-32618) (Invitrogen, Carlsband, CA, USA). After washing, tissues were dehydrated and counterstained with hematoxylin and mounted with DPX (Merck, Darmstadt, Germany). Negative controls were included, via omission of the primary antibodies.

### 2.6. Protein Analysis

For Western blot analysis, kidneys were immediately frozen with liquid nitrogen and stored at −80 °C. Kidney proteins were extracted through homogenization via ultrasonication, using ice-cold RIPA buffer. The homogenates were centrifuged, and the protein concentration of the supernatant was assayed using the bicinchoninic acid (BCA) protein assay kit (Pierce™ BCA, ThermoScientific, Rockford, IL, USA).

Aliquots of the extract containing 150–200 µg of proteins were separated via electrophoresis, using 12% SDS-PAGE gels and, afterwards, were transferred into nitrocellulose membranes. Membranes were blocked with 5% non-fat milk and incubated overnight at 4 °C with the same primary antibodies used for immunohistochemistry analysis. Thereafter, membranes were washed and incubated with anti-rabbit secondary antibody (1:1000) HRP-conjugated (sc-2004, Santa Cruz Biotechnology Inc., Dallas, TX, USA). Immunoreactive proteins were detected using the enhanced chemiluminescence method (ECL; WesternBright, Advansta, San Jose, CA, USA), and visualized on a ChemiDoc™ Imaging System (Bio-Rad Laboratories). Finally, the optical density of the bands was determined using Image Lab™ Software (Bio-Rad Laboratories, Hercules, CA, USA). Results were normalized against beta-Actin (1:500, SICGEN, Cantanhede, Portugal) concentrations and an internal control was loaded in all gels.

### 2.7. Statistical Analysis

Descriptive statistics are presented as mean ± standard errors of the mean (SEM) or as a count with percentages for categorical variables. Comparison between groups were performed using Kruskal–Wallis or one-way ANOVA. The strength of the association between continuous variables was estimated using Spearman’s correlation coefficient. Statistical significance was accepted at *p* < 0.05. Statistical analysis was performed using the IBM Statistical Package for Social Sciences (SPSS) for Windows, version 26.0 (IBM, Armonk, NY, USA) or GraphPad Prism^®^ software, version 9.4.1 (GraphPad Software, Inc., San Diego, CA, USA).

## 3. Results

### 3.1. Body and Kidney Weight

At the beginning of the study, all groups showed a similar body weight (BW). During the 5-week protocol, BW variation was negative in animals with moderate CRF, while the other study groups showed a similar increase in BW (Table 1).

At the end of the protocol, we observed a significant increase in the right kidney weight (KW), as well as in the relative right KW in the mild CRF group, when compared to the sham group. Despite having just 1/3 of the left kidney, the left KW and relative left KW in the moderate CRF group were similar to sham (Table 1).

### 3.2. Hematological and Renal Function Data

The moderate CRF group presented a consistent decrease in RBC count, hemoglobin and hematocrit values, compared to the sham group. However, only the reduction in hemoglobin concentration reached statistical significance (Table 2). Reticulocyte count and percentage, as well as RPI, were significantly decreased in rats with moderate CKD, compared to rats with mild disease.

Serum and urinary renal function data are illustrated in Table 3. At the end of the protocol, there were no significant differences regarding serum and urine measures of creatinine and urea, as well as concerning their clearances, between the sham and mild CRF group. However, all renal function parameters evaluated were aggravated in the moderate CRF group versus the sham group. Even though there was a steady decrease in eGFR alongside CKD severity, only the moderate CRF group’s decline was significant, compared to sham.

### 3.3. Kidney Histomorphology

Glomerular and tubulointerstitial lesions were evaluated and quantified (Supplementary Tables S1 and S2, respectively) in kidney sections stained with PAS.

At the end of the protocol, we observed the existence of glomerular crescent-like structures in the sham group, a feature that was common to all group animals (8/8) at a similar rate of frequency. These structures were previously identified as an early signature of nephropathy, in a rat model of prediabetes [30], but no significant changes between groups were relevant. The overall observation suggested the absence of any other changes in both glomerular and tubulointerstitial structures, despite the presence of inflammatory infiltrate in one out of eight of the sham operated animals, without signs of interstitial fibrosis and atrophy.

**Figure 1:**
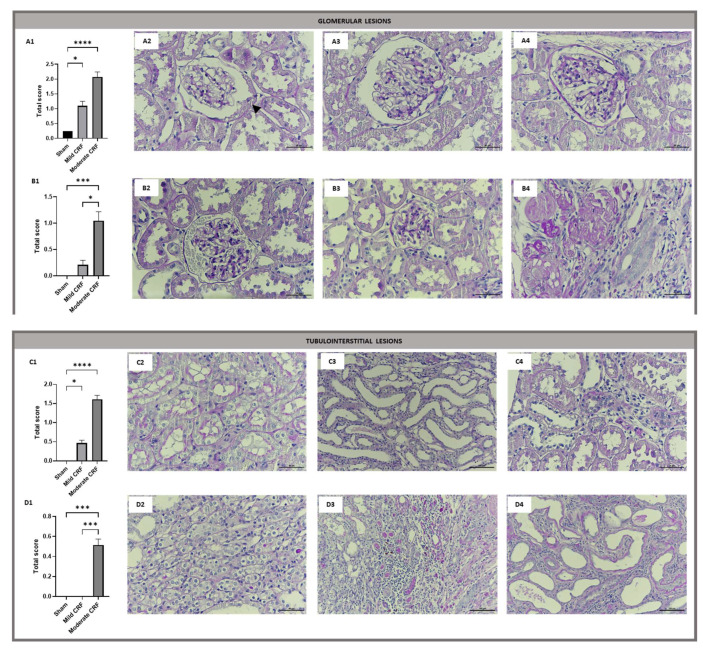
Total score of glomerular and tubulointerstitial lesions and representative images of the renal lesions observed in rats under study, at the end of the protocol (PAS staining, 400×) The scale bar indicated in each image represents 50 µm. (**A1**), total score for mild glomerular lesions; (**A2**), crescent-like structure (black arrows); (**A3**), glomerular hypertrophy and dilatation of Bowman’s space; (**A4**), glomerular basement membrane thickening; (**B1**), total score for advanced glomerular lesions; (**B2**), glomerular “blebbing”; (**B3**), glomerular atrophy; (**B4**), glomerulosclerosis; (**C1**), total score for mild tubulointerstitial lesions; (**C2**), hydropic degeneration; (**C3**), tubular dilatations, (**C4**), interstitial inflammatory infiltrate; (**D1**), total score for advanced tubulointerstitial lesions; (**D2**), atrophic tubules with irregular basement membranes; (**D3**), hyaline cylinders and moderate tubulointerstitial lesions; (**D4**), interstitial fibrosis and severe tubular dilatations with epithelial atrophy. Results are presented as mean ± standard error of the mean (SEM). The Kruskal–Wallis or one-way ANOVA test were used to compare groups, followed by Bonferroni correction for multiple comparisons (* *p* < 0.05, *** *p* < 0.001, **** *p* < 0.0001).

Regarding the mild CRF group, several mild glomerular and tubulointerstitial lesions were found: all rats (n = 8) presented some rate of glomerular hypertrophy, dilatation of Bowman’s space and tubular atrophy; however, a significantly increased total score was only observed for mild glomerular lesions, when compared to the sham group. Some advanced glomerular lesions (Figure 1(B1–B4)) were also found in mild CRF, but no significant changes were found in the total score, when compared to sham.

The moderate CRF group presented the highest total score of all kidney lesions under study. All rats of this group (n = 7) presented with mild glomerular and tubulointerstitial lesions, such as glomerular basement membrane thickening, glomerular hypertrophy and dilatation of Bowman’s space. Advanced glomerular lesions, including glomerular “blebbing” (4/7), glomerular atrophy (7/7) and glomerulosclerosis (7/7) were found in the moderate CRF rats. In addition, the moderate CRF group presented hyaline cylinders (4/7), vacuolar tubular degeneration (4/7), as well as interstitial fibrosis and tubular atrophy (IFTA) (7/7), which were absent in all other groups. None of the rats under study presented tubular calcification or necrosis.

### 3.4. Inflammatory Markers

**Figure 2:**
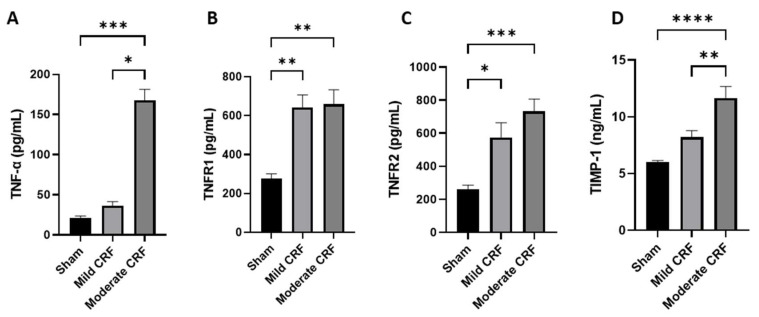
Circulating levels of TNF-α (**A**), TNFR1 (**B**), TNFR2 (**C**) and TIMP-1 (**D**) in sham (n = 8), mild CRF (n = 8) and moderate CRF (n = 7) groups. Results are presented as mean ± S.E.M. The Kruskal–Wallis or one-way ANOVA test were used to compare groups, followed by Bonferroni correction for multiple comparisons (* *p* < 0.05, ** *p* < 0.01, *** *p* < 0.001, **** *p* < 0.0001).

Both TNFR1 and TNFR2 showed significantly higher values for the earlier disease stage, the mild CRF group, compared to controls, while the other markers, TNF-α and TIMP-1, presented similar values. Moreover, the serum levels of TNFR2 and TIMP-1 increased with renal function worsening, but only TIMP-1 reached statistical significance (Figure 2). Actually, for both TNF-α and TIMP-1, the highest circulating levels were observed in the more advanced disease stages, showing significantly higher values than those presented by the sham and mild CRF groups.

The TNFR2 serum levels were negatively and significantly correlated with eGFR (r = −0.506, *p* = 0.014) and positively with serum creatinine (r = 0.675, *p* < 0.01).

Mild and advanced glomerular and tubulointerstitial lesions observed were significantly and negatively correlated with eGFR, and significantly and positively correlated with serum levels of all biomarkers, except for TNFR1, which showed no correlation with advanced tubular lesions.

Supplementary Figures S1–S4 show the differences in the circulating levels of each biomarker by each histopathologic lesion evaluated. Serum TNFR1 and TNFR2 levels increase with glomerular hypertrophy, dilatation of Bowman space and tubular atrophy (*p* < 0.01 for all). However, in more advanced lesions, such as glomerulosclerosis and IFTA, an inverse tendency was observed. No other relevant and clear differences were found in this analysis.

**Figure 3:**
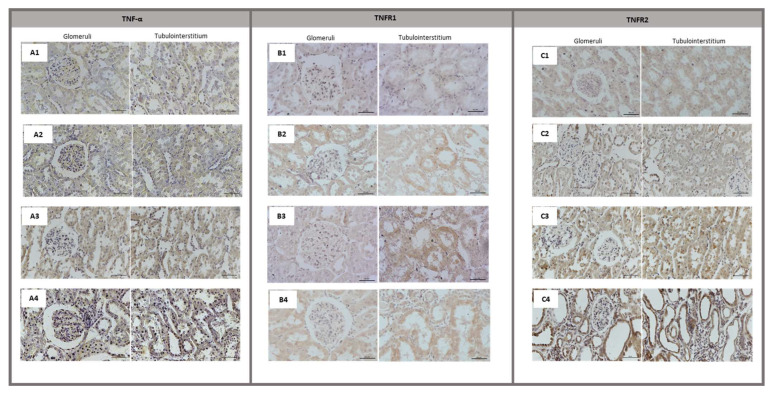
Renal TNF-α, TNFR1 and TNFR2 immunostaining. Representative images of immunostaining for TNF-α, TNFR1 and TNFR2 in the renal cortex and medulla of rat groups under study (400×). The scale bar indicated in each image represents 50µm. (**A1**), negative reaction for TNF-α; (**A2**), TNF-α immunostaining in sham group; (**A3**), TNF-α immunostaining in mild CRF group; (**A4**), TNF-α immunostaining in moderate CRF group; (**B1**), negative reaction for TNFR1; (**B2**), TNFR1 immunostaining in sham group; (**B3**), TNFR1 immunostaining in mild CRF group; (**B4**), TNFR1 immunostaining in moderate CRF group; (**C1**), negative reaction for TNFR2; (**C2**), TNFR2 immunostaining in sham group; (**C3**), TNFR2 immunostaining in mild CRF group; (**C4**), TNFR2 immunostaining in moderate CRF group.

No staining was observed on kidney sections when the primary antibodies were omitted (Figure 3(A1,B1,C1)). In the histologically normal kidney, there was occasional weak immunostaining for TNFR1 (Figure 3(B2)) and TNFR2 (Figure 3(C2)) on epithelial tubular cells, but in general, no TNF receptors were detected on renal tubules or glomeruli.

TNF-α immunostaining in glomeruli and tubulointerstitium was negative for both sham rats and mild CRF rats, compared with the negative controls (Figure 3(A1–A3)). In the 5/6 nephrectomy group, the labelling was strongly positive for TNF-α (Figure 3(A4)). A moderate signal for TNFR1 was only evident in tubular epithelial cells, in both CRF groups (Figure 3(B3,B4)). Considering TNFR2 immunolabeling, we found an increased intensity on renal tubules with a worsening disease (Figure 3(C3,C4)).

**Figure 4:**
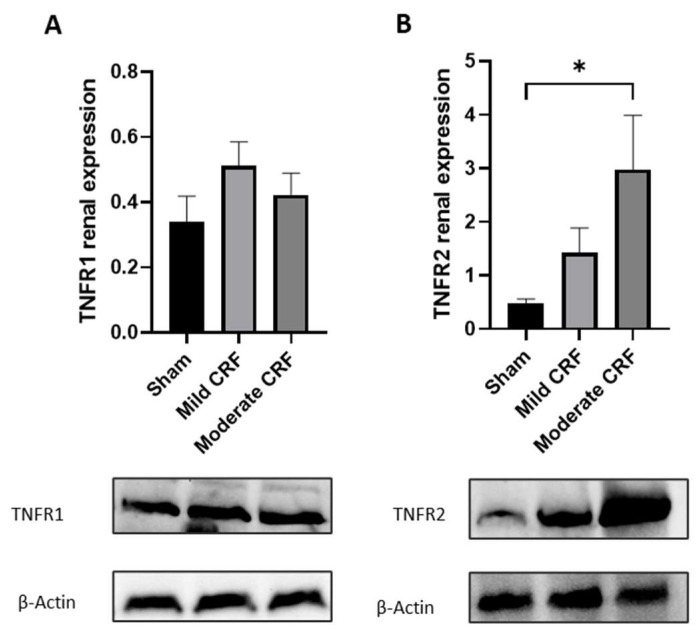
Evaluation of renal expression of TNFR1 (**A**) and TNFR2 (**B**) from Western blotting and representative images for each study group. Results are presented as mean ± S.E.M. The Kruskal–Wallis or one-way ANOVA test were used to compare groups, followed by Bonferroni correction for multiple comparisons (* *p* < 0.05).

These results from the immunohistochemistry were supported by Western blotting analysis (Figure 4). Accordingly, there was a steady increase in TNFR2 renal expression, but only reaching statistical significance in moderate disease, when compared to controls. No significant changes in TNFR1 renal expression were detected.

TNFR2 renal expression levels showed correlations with the score of mild and advanced tubular lesions (r = 0.501, *p* = 0.015 and r = 0.5518, *p* = 0.011, respectively), and also with eGFR (r = −0.442, *p* = 0.035).

## 4. Discussion

During the last few decades, several advances have occurred in the management of CKD patients; however, morbidity and mortality rates are still high, compared to the general population [1,31]. Chronic inflammation is the driving force in the progression of kidney diseases and play a key role in the development of its associated comorbidities [32,33]. The inflammatory process begins in the early stages of the disease, regardless of its cause, eventually leading to renal fibrosis and end-stage renal disease (ESRD) [33]. In this study, we evaluated several inflammatory markers related to TNF signaling (TNF-α, TNFR1 and TNFR2) and the inhibition of metalloproteinases (TIMP-1), and analyzed kidney histopathologic lesions in rats with mild and moderate disease.

After 5 weeks of nephrectomy, the mild CRF group rats developed an early degree of renal insufficiency. The surgical removal of the left kidney induced a compensatory hypertrophy of the right kidney, confirmed by the increased RKW/BW ratio, which resulted in non-significant alterations of serum and urinary measures of renal function, and by the absence of anemia. However, several mild glomerular and tubulointerstitial lesions were found, with a significantly increased total score only for mild glomerular lesions, when compared to the sham group. The absence of differences in creatinine, and subsequently eGFR, is a known limitation of these classical CKD biomarkers in early disease detection, since it does not change until kidney function is substantially impaired [34,35]. The surgical 5/6 nephrectomy model is a well-established model of moderate, but sustained CKD [36]. Despite having only 1/3 of the left kidney, the moderate CRF group showed a trend to increased KW, consistent with a compensational status, since the ratio of KW/BW was similar to the sham group. This increase in KW was accompanied by a deterioration in kidney function, as we found a significant increase in serum urea and creatinine, alongside decreased urinary levels and clearance, as well as decreased eGFR. Additionally, several advanced renal lesions were observed in both glomeruli and the tubulointerstitial area. In this group, the negative change in BW may be related to a loss of appetite, due to uremic milieu and anemia. Anemia is a known complication of CKD and results primarily from decreased erythropoietin synthesis in the kidney. This is in line with our results, demonstrating a lower hemoglobin concentration and a reduction in RPI in moderate CKD.

The TNF-α signaling pathway, through its receptors TNFR1 and TNFR2, seems to be essential in renal function deterioration [37]. However, the mechanisms through which the TNFRs initiate and perpetuate renal damage are not completely understood [38]. In addition to the various studies addressing the association of TNFRs with human kidney disease and related clinical outcomes [18,39,40,41], several animal studies highlight their role in the pathophysiology of kidney diseases [24,25,42].

In clinical studies, soluble TNF receptors 1 and 2 were showed to be increased in several cohorts of diabetic patients. Accordingly, our group also reported increased levels of TNFR2 in patients with decreased eGFR and suggested their potential as a biomarker of early CKD, as well as disease staging/worsening in a cohort of CKD patients with diverse etiologies [14]. We found a similar pattern for this biomarker in the present study. The circulating levels of both TNFR1 and TNFR2 were significantly increased in early disease, the mild CRF group, while the other markers, TNF-α and TIMP-1, were not increased at this stage. However, only TNFR2 serum levels seem to increase steadily with the worsening of renal function, as documented by the inverse correlation with eGFR.

A steady increase in TNFR2 renal expression was also found, but only reaching statistical significance in moderate disease, when compared to controls, and no significant changes in TNFR1 renal expression were detected. In addition, TNFR2 renal expression showed correlations with the score of mild and advanced tubular lesions. These differences are likely explained by the different biological actions of the two receptors, which engage shared and different downstream signaling pathways [37]. In accordance with our findings, renal TNFR2 expression, not TNFR1, seems to mediate the development of glomerulonephritis [24] and cisplatin-induced acute renal failure [42] in mice models. As reported by Al-Lamki et al., TNFR1 seems to be basally expressed in the normal kidney, while TNFR2 expression is upregulated after renal injury and is also mostly expressed in immune cells [43]. TNFR1 is mostly implicated in tissue inflammation and injury; however, TNFR2 promotes epithelial-to-mesenchymal transition and cell proliferation, and has been mainly associated with the nuclear factor kappa B (NF-кB) pathway, being implicated in immune modulation and tissue regeneration [44]. As we previously hypothesized [14], the greater expression of renal *TNFR2* by the activated leukocytes and injured renal cells activates the NF-кB mediated pathway, through TNF-α signaling, to promote injury resolution. Thus, TNFR2 seems to act not only as an early biomarker of renal damage but also as a mediator of the disease.

Furthermore, we hypothesized that these inflammatory biomarkers might correlate with histological findings, since most of the published literature on TNFR1 and TNFR2 has focused on CKD patients without biopsy confirmation. Our cross-sectional analysis showed that serum levels of TNFR1 and TNFR2 are correlated with the total score of mild glomerular lesions, particularly glomerular hypertrophy and dilatation of Bowman’s space, and also tubular atrophy. Plasma TNFR1 and TNFR2 have been previously associated with early glomerular lesions in type 2 diabetic patients [45] and with tubulointerstitial lesions in patients across a diverse set of kidney diseases [39,46]. However, Niewczas et al. reported no correlations of renal mRNA expression of TNFR1 and TNFR2 in advanced lesions, such as glomerular sclerosis and IFTA, in patients with diabetic nephropathy [40].

The diverse range of kidney diseases, the prevalence and complexity of associated comorbidities, and the multiple drug therapies are major obstacles when assessing inflammatory parameters in CKD patients. In this study, we used rat models of mild and moderate CKD, which allowed us to study these disease stages without the heterogeneity and individual variability that is observed in CKD patients. However, our study has some limitations that should be addressed. First, the cross-sectional design does not allow us to fully characterize biomarkers with renal function deterioration. Additionally, the results were obtained with a specific model of CKD, and thus, might not be reproducible in other conditions.

In conclusion, our findings suggest that TNFR2 seems to play an early role in the renal cell damage underlying CKD development, and to have potential as biomarker for an earlier detection, as compared to classic biomarkers of renal (dys)function; moreover, it has potential as a biomarker of disease progression, as reflected by the circulating levels. Therefore, TNFR2 is indicated as a new therapeutic target and its blockade may be a promising strategy in the treatment of CKD. Further mechanistic studies would be necessary to understand the prognostic value of this biomarker.

## Figures and Tables

**Table 1:** Body and kidney weight of rat groups under study, at the end of the protocol.

	Sham (n = 8)	Mild CRF (n = 8)	Moderate CRF (n = 7)
**Body Weight (BW)**			
Initial T0 (g)	319.63 ± 6.05	307.63 ± 9.09	352.29 ± 30.88
Final T36 (g)	371.88 ± 5.52	360.63 ± 3.57	348.57 ± 15.22
∆BW	52.25 ± 3.30	53.00 ± 7.74	−3.71 ± 25.37 *a*
**Kidney Weight (KW)**			
Left KW (g)	1.4813 ± 0.0561	-	1.5352 ± 0.0865
Left KW/BW (g/Kg)	3.99 ± 0.15		4.44 ± 0.27
Right KW (g)	1.5592 ± 0.0707	2.0277 ± 0.0608 *a*	-
Right KW/BW (g/Kg)	4.20 ± 0.19	5.62 ± 0.16 *a*	

Results are presented as mean ± S.E.M. The Kruskal–Wallis or one-way ANOVA test were used to compare groups, followed by Bonferroni correction for multiple comparisons (***a*** *p* < 0.05 vs. sham). ∆BW, body weight variation.

**Table 2:** Hematological data for rat groups under study, at the end of the protocol.

	Sham (n = 8)	Mild CRF (n = 8)	Moderate CRF (n = 7)
RBC (10¹^2^/µL)	8.16 ± 0.19	8.21 ± 0.27	7.71 ± 0.38
Hb (g/L)	152 ± 37	151 ± 33	137 ± 51 *a*
Ht (L/L)	0.46 ± 0.01	0.46 ± 0.01	0.42 ± 0.02
WBC (10⁹/L)	5.21 ± 0.55	5.46 ± 0.40	4.46 ± 0.54
RET (10⁹/L)	25.00 ± 0.9	28.2 ± 1.6	20.7 ± 1.5 *b*
% RET	3.073 ± 0.117	3.460 ± 0.213	2.701 ± 0.222 *b*
RPI	3.073 ± 0.126	3.481 ± 0.199	2.443 ± 0.191 *b*
Neut (10⁹/L)	0.93 ± 0.17	0.81 ± 0.17	0.82 ± 0.12
Lym (10⁹/L)	4.20 ± 0.38	4.57 ± 0.30	3.55 ± 0.48
NLR	0.210 ± 0.027	0.175 ± 0.031	0.249 ± 0.041
PLT (10⁹/L)	672 ± 27	674 ± 30	704 ± 41

Results are presented as mean ± S.E.M. The Kruskal–Wallis or one-way ANOVA test were used to compare groups, followed by Bonferroni correction for multiple comparisons (***a*** *p* < 0.05 vs. sham; ***b*** *p* < 0.05 vs. mild CRF). Hb, hemoglobin; Ht, hematocrit; Lym, lymphocytes; Neut, neutrohpils; NLR, neutrophil-to-lymphocyte ratio; PLT, platelets; RBC, red blood cells; RET, reticulocytes; RPI, reticulocyte production index; WBC, white blood cells.

**Table 3:** Serum and urinary renal function data of rat groups under study, at the end of the protocol.

	Sham (n = 8)	Mild CRF (n = 8)	Moderate CRF (n = 7)
**Creatinine**			
Serum (mg/dL)	0.41 ± 0.01	0.48 ± 0.02	1.26 ± 0.44 ***a***
Urine (mg/dL)	74.90 ± 6.84	82.87 ± 11.81	39.26 ± 10.94 ***b***
Clearance (mL/h)	112.88 ± 7.60	95.88 ± 4.11	57.86 ± 14.72 ***ab***
**Urea**			
Serum (mg/dL)	37.75 ± 1.99	49.13 ± 2.15	123.29 ± 37.92 ***a***
Urine (mg/dL)	4281 ± 325	4497 ± 356	2146 ± 549 ***ab***
Clearance (mL/h)	70.13 ± 4.24	53.38 ± 4.08	40.29 ± 8.20 ***a***
**eGFR (mL/h)**	91.50 ± 5.79	74.63 ± 3.89	48.71 ± 10.76 ***a***

Results are presented as mean ± S.E.M. The Kruskal–Wallis or one-way ANOVA test were used to compare groups, followed by Bonferroni correction for multiple comparisons (***a*** *p* < 0.05 vs. sham; ***b*** *p* < 0.05 vs. mild CRF). eGFR, estimated glomerular filtration rate.

## Data Availability

Additional supporting information may be provided by the corresponding authors upon reasonable request.

## Supplementary Materials

The following supporting information can be downloaded at: https://www.mdpi.com/article/10.3390/biom13030534/s1, Table S1: Histopathologic features of rat groups under study, at the end of protocol and Table S2: Total score of mild and avnaced kidney lesions of rat groups under study, at the end of protocol; Figure S1: Association of mild glomerular lesions with the circulating levels of TNF-α, TNFR1, TNFR2 and TIMP-1, Figure S2: Association of advanced glomerular lesions with the circulating levels of TNF-α, TNFR1, TNFR2 and TIMP-1, Figure S3: Association of mild tubulointerstitial lesions with the circulating levels of TNF-α, TNFR1, TNFR2 and TIMP-1 and Figure S4: Association of advanced tubulointerstitial lesions with the circulating levels of TNF-α, TNFR1, TNFR2 and TIMP-1.
