# Supplementary material for: TNFR2 as a Potential Biomarker for Early Detection and Progression of CKD
Source: Biomolecules. 2023 Mar 15;13(3):534. doi: 10.3390/biom13030534 (PMC10046457; doi:10.3390/biom13030534)
Supplement: Supplementary file 1 [file biomolecules-13-00534-s001.zip › Supplementary Figures S1, S2, S3 and S4.pdf]

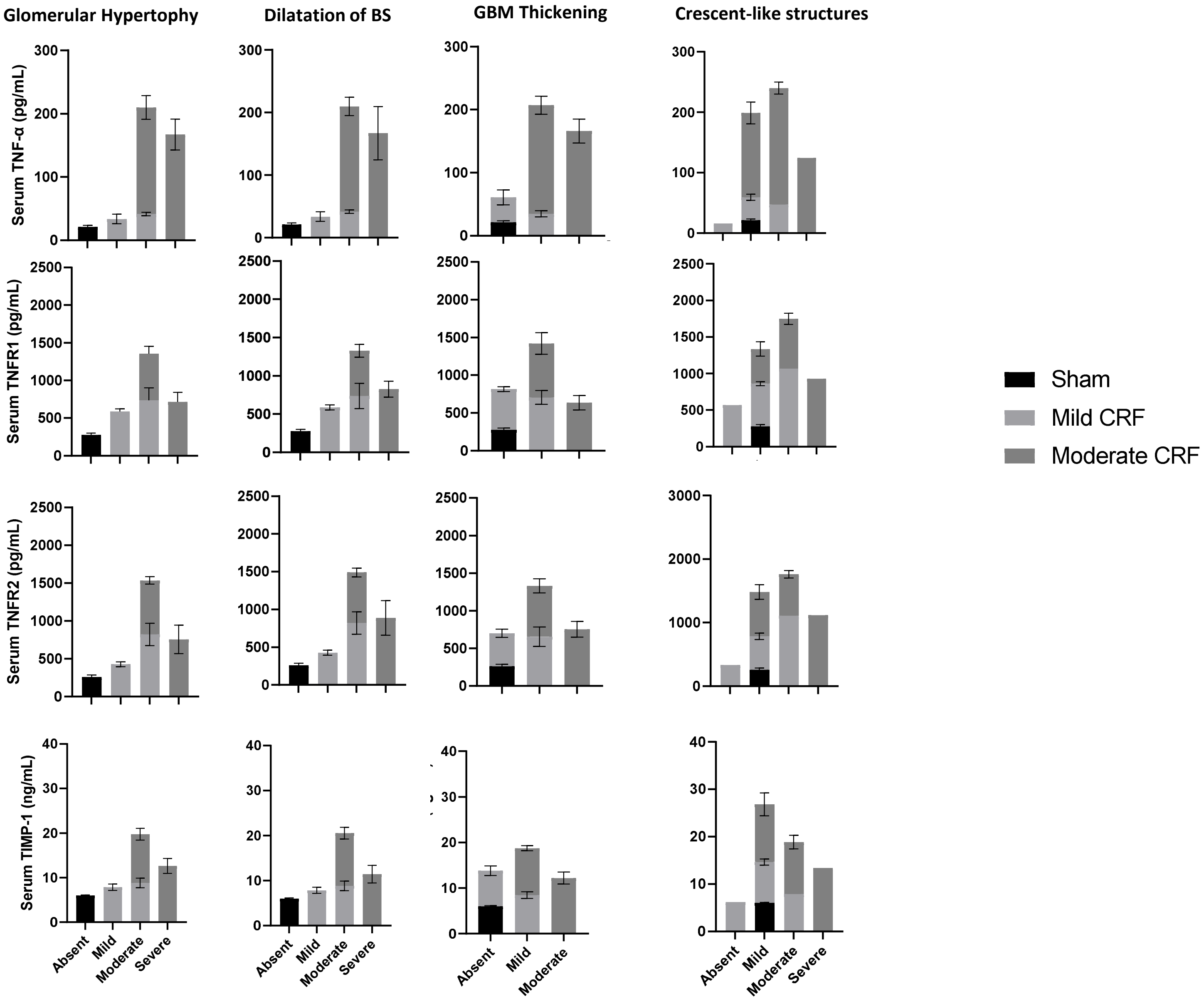

Supplementary Figure S1: Association of mild glomerular lesions with the circulating levels of TNF-α, TNFR1, TNFR2 and TIMP-1

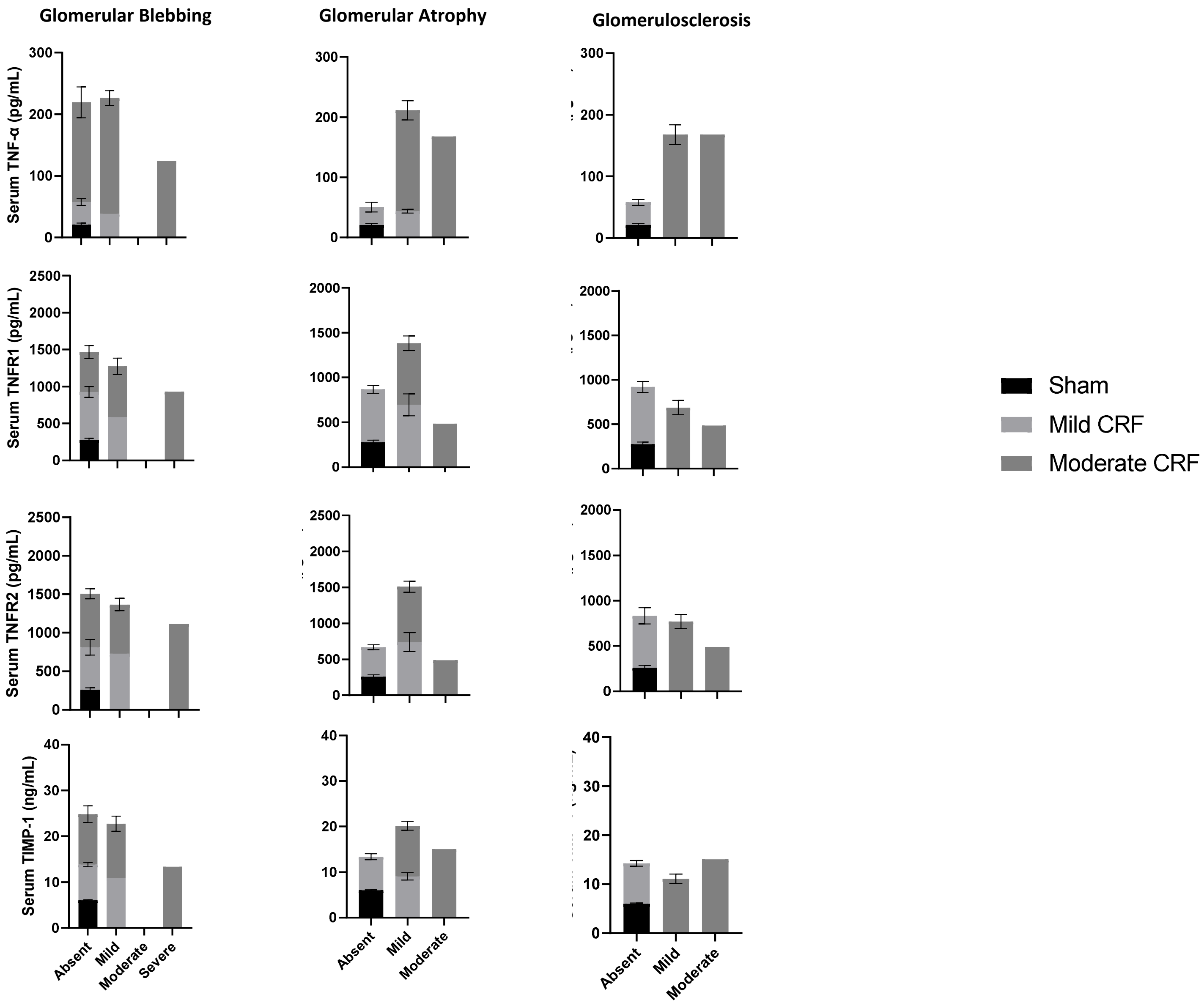

Supplementary Figure S2: Association of advanced glomerular lesions with the circulating levels of TNF-α, TNFR1, TNFR2 and TIMP-1

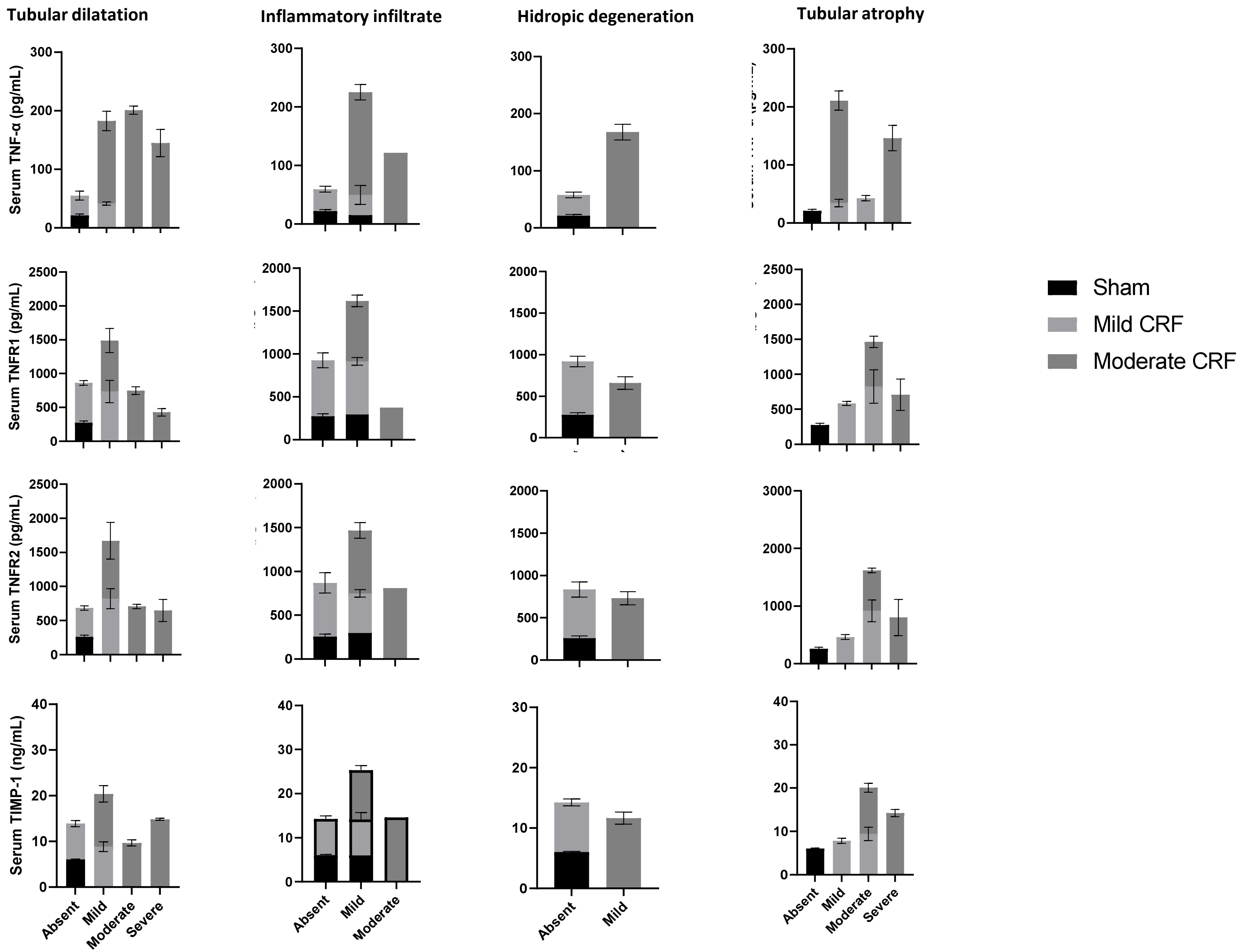

Supplementary Figure S3: Association of mild tubulointerstitial lesions with the circulating levels of TNF-α, TNFR1, TNFR2 and TIMP-1

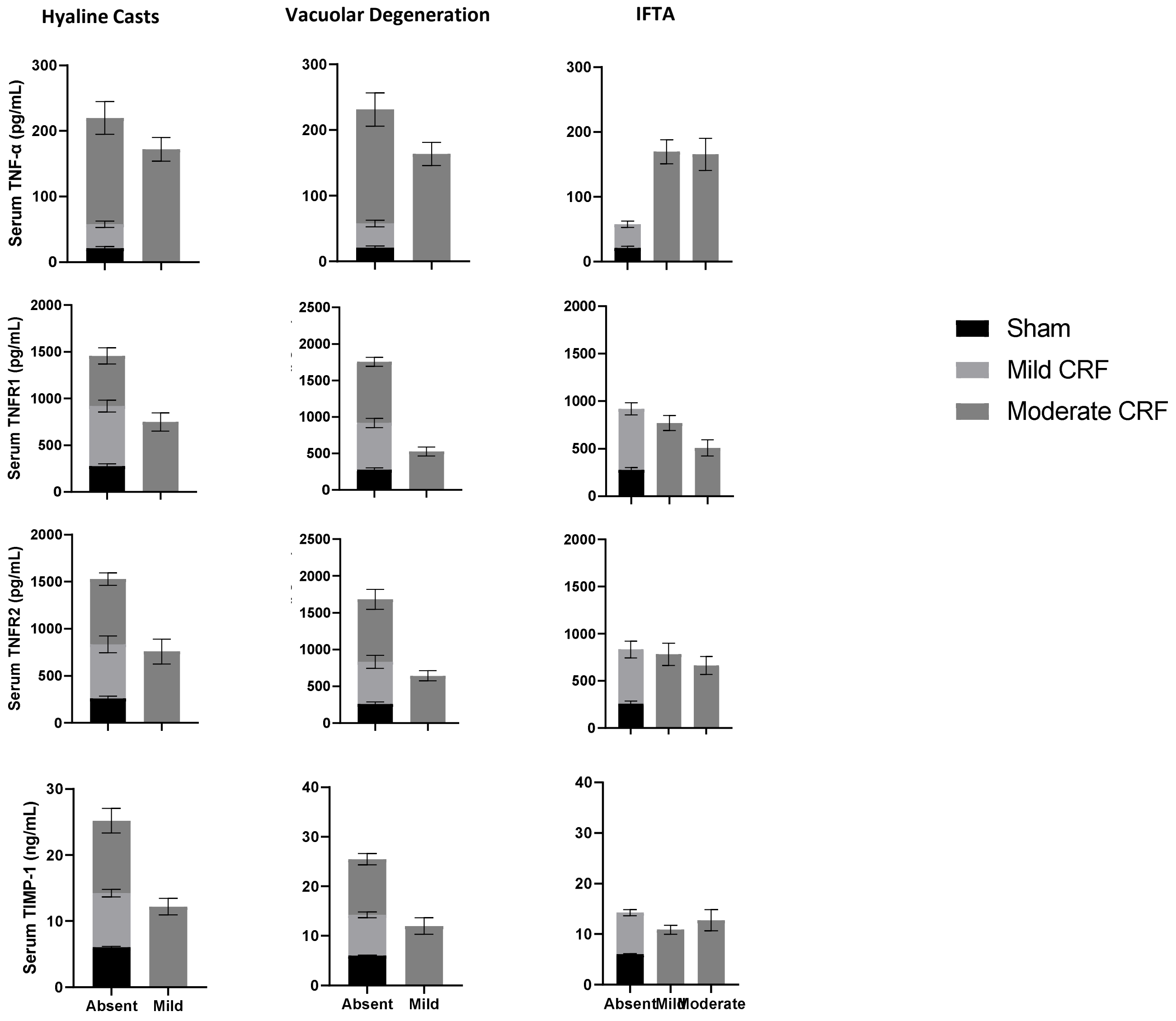

Supplementary Figure S4: Association of advanced tubulointerstitial lesions with the circulating levels of TNF-α, TNFR1, TNFR2 and TIMP-1
