# Supplementary material for: TNFR2 as a Potential Biomarker for Early Detection and Progression of CKD
Source: Biomolecules. 2023 Mar 15;13(3):534. doi: 10.3390/biom13030534 (PMC10046457; doi:10.3390/biom13030534)
Supplement: Supplementary file 1 [file biomolecules-13-00534-s001.zip › Supplementary Tables S1 and S2.pdf]

***Supplementary Table S1. Histopathologic feautures of rat groups under study, at the end of protocol***

| | (Total n= 23) | | Sham (n = 8) | | CKD 1-2 (n = 8) | | CKD 3-4 (n = 7) | | ***P*** |
|---|---|---|---|---|---|---|---|---|---|
| | **Absent n (%)** | **Present n (%)** | **Absent** | **Present** | **Absent** | **Present** | **Absent** | **Present** | |
| ***Mild Glomerular Lesions*** | | | | | | | | | |
| Glomerular Hypertrophy | 8 (34.8) | 15 (65.2) | 8 (100) | 0 | 0 | 8 (100) | 0 | 7 (100) | ***< 0.001*** |
| Crescent-like structures | 1 (4.3) | 22 (95.7) | 0 | 8 (100) | 1 (12.5) | 7 (87.5) | 0 | 7 (100) | 0.375 |
| Dilatation of Bowman's space | 8 (34.8) | 15 (65.2) | 8 (100) | 0 | 0 | 8 (100) | 0 | 7 (100) | ***< 0.001*** |
| GBM Thickening | 11 (47.8) | 12 (52.2) | 8 (100) | 0 | 3 (37.5) | 5 (62.5) | 0 | 7 (100) | ***< 0.001*** |
| ***Advanced Glomerular Lesions*** | | | | | | | | | |
| Glomerular "Blebbing" | 18 (78.3) | 5 (21.7) | 8 (100) | 0 | 7 (87.5) | 1 (12.5) | 3 (42.9) | 4 (57.1) | ***0.02*** |
| Glomerular Atrophy | 12 (52.2) | 11 (47.8) | 8 (100) | 0 | 4 (50.0) | 4 (50.0) | 0 | 7 (100) | ***< 0.001*** |
| Glomerulosclerosis | 16 (69.6) | 7 (30.4) | 8 (100) | 0 | 8 (100) | 0 | 0 | 7 (100) | ***< 0.001*** |
| ***Mild Tubulointerstitial Lesions*** | | | | | | | | | |
| Tubular dilatation | 13 (56.5) | 10 (43.5) | 8 (100) | 0 | 5 (62.5) | 3 (37.5) | 0 | 7 (100) | ***< 0.001*** |
| Interst. Inflamm. Infilt. | 13 (56.5) | 10 (43.5) | 7 (87.5) | 1 (12.5) | 6 (75.0) | 2 (25.0) | 0 | 7 (100) | ***< 0.001*** |
| Hidropic Tubular Degen | 16 (69.6) | 7 (30.4) | 8 (100) | 0 | 8 (100) | 0 | 0 | 7 (100) | ***< 0.001*** |
| Tubular Atrophy | 8 (34.8) | 15 (65.2) | 8 (100) | 0 | 0 | 8 (100) | 0 | 7 (100) | ***< 0.001*** |
| ***Advanced Tubulointerstitial Lesions*** | | | | | | | | | |
| Hyaline Cylinders | 19 (82.6) | 4 (17.4) | 8 (100) | 0 | 8 (100) | 0 | 3 (42.9) | 4 (57.1) | ***0.004*** |
| Tubular Calcification | 23 (100) | 0 | 8 (100) | 0 | 8 (100) | 0 | 7 (100) | 0 | - |
| Vacuolar Tubular Degen | 19 (82.6) | 4 (17.4) | 8 (100) | 0 | 8 (100) | 0 | 3 (42.9) | 4 (57.1) | ***0.004*** |
| IFTA | 16 (69.6) | 7 (30.4) | 8 (100) | 0 | 8 (100) | 0 | 0 | 7 (100) | ***< 0.001*** |
| Tubular Necrosis | 23 (100) | 0 | 8 (100) | 0 | 8 (100) | 0 | 7 (100) | 0 | - |

Results expressed as n (%)

Pearson's chi-square test was used to compare observed and expected frequencies

*P* -value: Absence of lesion vs Presence of lesion

BS, Bowman's space; Degen., degeneration; GBM, glomerular basement membrane; IFTA, interstitial fibrosis and tubular atrophy; Inters. Inflamm. Infilt., interstitial inflammatory filtration.

***Suplementary Table S2. Total score of mild and avnaced kidney lesions of rat groups under study, at the end of protocol***

| | Sham (n = 8) | CKD 1-2 (n = 8) | CKD 3-4 (n = 7) |
|---|---|---|---|
| **Mild Glomerular Lesions** | | | |
| Glomerular Hypertrophy | 0 | 1.38 ± 0.18 ***a*** | 2.43 ± 0.20 ***a*** |
| Crescent-like structures | 1.00 ± 0.00 | 1.00 ± 0.19 | 1.86 ± 0.26 ***ab*** |
| Dilatation of Bowman's space | 0 | 1.38 ± 0.18 ***a*** | 2.29 ± 0.18 ***a*** |
| GBM Thickening | 0 | 0.63 ± 0.18 | 1.71 ± 0.18 ***a*** |
| **Total** | 0.25 ± 0.00 | 1.09 ± 0.16 ***a*** | 2.07 ± 0.17 ***a*** |
| **Advanced Glomerular Lesions** | | | |
| Glomerular "Blebbing" | 0 | 0.13 ± 0.13 | 0.86 ± 0.40 ***a*** |
| Glomerular Atrophy | 0 | 0.50 ± 0.19 | 1.14 ± 0.14 ***a*** |
| Glomerulosclerosis | 0 | 0 | 1.14 ± 0.14 ***ab*** |
| **Total** | 0 | 0.21 ± 0.09 | 1.05 ± 0.17 ***ab*** |
| **Mild Tubular Lesions** | | | |
| Tubular dilatation | 0 | 0.38 ± 0.18 | 2.00 ± 0.31 ***ab*** |
| Inters. Inflamm. Infilt. | 0.13 ± 0.13 | 0.25 ± 0.16 | 1.14 ± 0.14 ***ab*** |
| Hidropic Tubular Degen. | 0 | 0 | 1.00 ± 0.00 ***ab*** |
| Tubular Atrophy | 0 | 1.25 ± 0.16 ***a*** | 2.29 ± 0.18 ***a*** |
| **Total** | 0 | 0.47 ± 0.07 | 1.61 ± 0.11 ***a*** |
| **Advanced Tubular Lesions** | | | |
| Hyaline Cylinders | 0 | 0 | 0.57 ± 0.20 ***ab*** |
| Tubular Calcification | 0 | 0 | 0 |
| Vacuolar Tubular Degen. | 0 | 0 | 0.57 ± 0.20 ***ab*** |
| IFTA | 0 | 0 | 1.43 ± 0.20 ***ab*** |
| Tubular Necrosis | 0 | 0 | 0 |
| **Total** | 0 | 0 | 0.51 ± 0.06 ***ab*** |

Results are presented as mean ± SEM.

The Kruskal-Wallis test was used to compare groups, followed by Bonferroni correction for multiple comparisons.
